# Hemophagocytic lymphohistiocytosis in the setting of HELLP Syndrome

**DOI:** 10.1002/ccr3.1828

**Published:** 2018-11-05

**Authors:** Sarmen Sarkissian, Yasir Khan, Daniel Farrell, David Constable, Elizabeth Brem

**Affiliations:** ^1^ Pacific Shoes Medical Group Long Beach California; ^2^ Division of Hematology and Oncology, Department of Medicine University of California, Irvine Irvine California; ^3^ Department of Pathology University of California, Irvine Irvine California; ^4^ Division of Infectious Disease, Department of Medicine University of California, Irvine Irvine California

**Keywords:** EBV, HELLP syndrome, hemophagocytic lymphohistiocytosis, hemophagocytosis

## Abstract

Hemophagocytic lymphohistiocytosis (HLH) is a life‐threatening hyper activation of the immune system. Rare cases associated with HELLP syndrome and other similar conditions in pregnancy have been reported. Despite the improved survival rates with etoposide and dexamethasone‐based regimens, HLH remains a challenging disease. Experience in pregnant patients is exceedingly rare.

## 1 INTRODUCTION

A 30‐year‐old woman (G1P0) presented at 35 weeks' gestation with fever and jaundice. Fever persisted after delivery, and she was diagnosed ultimately with hemophagocytic lymphohistiocytosis (HLH). Her outcome was poor despite aggressive therapy. Reports of HLH in the setting of HELLP syndrome and acute fatty liver of pregnancy are rare.

Hemophagocytic lymphohistiocytosis occurs when over‐reactive macrophages and histiocytes engulf erythrocytes in the bone marrow and spleen. Natural killer (NK) cell dysfunction leads to excess cytokine production from macrophages and cytotoxic lymphocytes. This uncontrolled release of cytokines leads to a spectrum of clinical symptoms ranging from flu‐like symptoms to end organ damage.^1^, ^2^, ^3^

Hemophagocytic lymphohistiocytosis is generally categorized as a primary or secondary phenomenon. Primary HLH occurs due to genetic mutations in the NK cell granzyme‐generating pathway and is generally diagnosed in childhood or early adulthood. Secondary HLH occurs in the setting of infection, autoimmune disease, or malignancy.^4^ Cases of HLH in pregnancy have only rarely been reported in the medical literature (Table 1). In some cases of secondary HLH, treating the underlying etiology (ie, initiating appropriate chemotherapy for the underlying malignancy or starting immunosuppression for rheumatologic disease) will lead to resolution of the HLH. In other cases, specific therapy targeting the HLH is necessary. There is no consensus standard of care therapy for HLH. The pediatric community's HLH‐94 protocol established the role of etoposide and dexamethasone as the backbone of therapy for primary HLH.^5^ The HLH‐2004 protocol has cyclosporine added to the protocol; long‐term results are pending.^6^ There is also published experience with CHOP (cyclophosphamide, adriamycin, vincristine, prednisone) chemotherapy and anti‐thymocyte globulin with encouraging results from small, nonrandomized studies.^7^, ^8^ Left untreated, HLH is uniformly fatal.

**Table 1:** Summation of available case reports of HLH diagnosed during pregnancy

Publication	Presenting symptoms	Associated condition	Gestational age at diagnosis (wk)	Fetal outcome	Treatment	Patient outcome
Kerley et al^11^	Dyspnea; abdominal pain;	HELLP syndrome	22	Delivered	HLH‐2004^a^, followed by allogeneic BMT	Relapsed 11 mo post‐transplant and died
Ikeda et al^14^	Fever, anorexia, pancytopenia	EBV	11	Delivered at 37 4/7 weeks (abstract available in English, but paper in Japanese)	Etoposide and cyclosporine remain in remission 10 mo post treatment.	
Giard et al^12^	Viral illness	Kikuchi‐Fujimoto Lymphadenitis, AFLP	13	Spontaneous abortion	Etoposide with dexamethasone	Died on day 48 secondary to PE
Pawar et al^15^	Fever, fatigue	Visceral leishmania	24	Delivered	Dexamethasone and antibiotics	In remission at the time of publication
Samra et al^16^	Cough, fever	Idiopathic	16	Completed pregnancy	Dexamethasone, in remission	In remission at the time of publication
Tumian et al^17^	Jaundice, anemia	Idiopathic	38	Delivered	Dex, Cyclosporine, in remission	In remission at the time of publication
Klein et al^18^	Diarrhea, GI bleeding	EBV	30	C‐section at 31 wk	Steroids, Cyclosporine, etoposide	Died
Chmait et al^19^	Routine checkup	EBV	29	Delivery at 30 wk		Died with multi organ failure.
Yamaguchi et al^20^	Fever, cytopenia	HSV2	Mid gestation	Delivery	Failed steroids, remission with cyclosporin A	In remission at the time of publication
Hanaoka et al^21^	Fever, cytopenia	B‐cell lymphoma	23	Emergent C‐section	R‐CHOP^b^	In remission at the time of publication
Perard et al^22^	Fevers	SLE	22	Premature delivery	IVIG with high dose pulse steroids.	In remission at the time of publication
Chien et al^23^	Fever	Unclear	23	C‐section	Dexamethasone	in remission at the time of publication
Teng et al^24^	Fever, cytopenia	Autoimmune hemolytic anemia	23	Terminated pregnancy	Failed steroids	Remission post termination of pregnancy
Arewa et al^25^	Jaundice	HIV	21	Delivery	HAART^c^	In remission at the time of publication
Dunn et al^26^	Rash, fever, headache	Still disease	19	Delivery	High dose steroids	In remission at the time of publication
Shukla et al^27^	Fever x 2 wk	Unclear etiology	10	Spontaneous abortion	Failed steroids	Remission after spontaneous abortion
Mayama et al^28^	Fever, pancytopenia	Parvovirus B19	21	Delivered at 37 4/7 weeks	Steroids	In remission at the time of publication

a HLH‐2004 = etoposide, dexamethasone, cyclosporine.

b R‐CHOP = rituximab, cyclophosphamide, hydroxydaunorubicin (adrimycin), vincristine (Oncovin), prednisone; common first‐line regimen for B‐cell lymphomas

c Highly active antiretroviral therapy (HAART).

Acute fatty liver of pregnancy (AFLP) is a clinical diagnosis. It presents in the third trimester with nausea, vomiting, abdominal pain, and jaundice. Laboratories show elevated transaminases, alkaline phosphatase, and bilirubin. Differentiating between AFLP and HELLP syndrome (hemolysis, elevated liver enzymes, low platelets) can be challenging. Evidence of hemolysis is considered to be more consistent with HELLP syndrome. The mainstays of management for both conditions are maternal stabilization as well as delivery of the fetus.^9^, ^10^, ^11^, ^12^

In this report, we present a young woman who received appropriate interventions for HELLP syndrome but soon after delivery was diagnosed with HLH. The clinical assessment was that the HELLP syndrome triggered the HLH. Despite initiation of HLH therapy, she ultimately passed away. This case raised for us the question of whether HLH that arises in the context of pregnancy and/or HELLP syndrome represents a subset of patients with a particularly poor prognosis. In addition to her case, we present a review of the (limited) literature available on this topic.

## 2 CASE DESCRIPTION

A 30‐year‐old G1P0 woman at 35 weeks and 2 days of gestation presented to obstetric (OB) triage with uterine contractions. She had a history of atrial septal defect repair as a child but was otherwise healthy. Ten days prior to admission, the patient had a noted oral mucosal lesions concerning for HSV‐1 infection but declined treatment with oral acyclovir. Five days prior to admission, she presented to urgent care with flu‐like symptoms and a temperature of up to 38.9°C for 2 weeks. She was given a course of azithromycin for presumed community‐acquired pneumonia.

On presentation to OB triage, vitals were notable for a temperature of 39.3°C and a heart rate of 105 beats per minute. Review of symptoms was positive for nausea and emesis. She was visibly jaundiced. Laboratories revealed alkaline phosphatase 591 U/L (normal 34‐104 U/L), total bilirubin 5.1 mg/dL (0‐1.4 mg/dL), aspartate aminotransferase (AST) 142 U/L (13‐39 U/L), hemoglobin 8.8 g/dL (11.5‐15 g/dL), platelets 67 000 (150‐400 × 10^3^/μL), and fibrinogen 547 mg/dL (215‐438 mg/dL). Haptoglobin was normal, 149 mg/dL (44‐215 mg/dL), suggesting that hemolysis was not actively occurring, so a presumptive diagnosis of AFLP was made. The patient underwent an emergent cesarian section (C‐section). A healthy male infant was delivered; to our knowledge, he was without any evidence of liver disease, particularly neonatal hemochromatosis. There was evidence of hypertrophic decimal vasculopathy in the placenta, which is seen in gestational hypertension (classically HEELP and pre‐eclampsia). This pathology helped retrospectively change the diagnosis of her the acute hepatic failure to HELLP as these vascular changes are not seen in AFLP.

**Figure 1:**
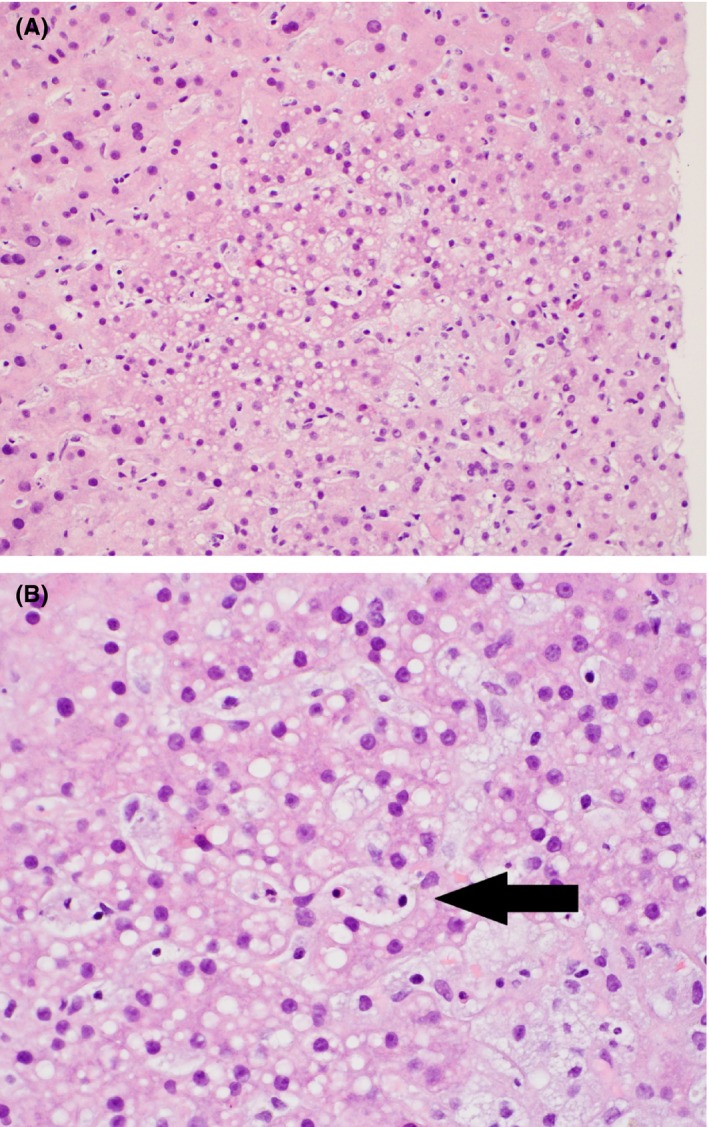
Liver biopsy at 20x (A) and 40x (B) magnifications. Hematoxylin and eosin (H&E) staining of the biopsy shows expanded portal tracts with hypertrophic Kupffer cells and patchy inflammatory infiltrates consisting of numerous macrophages, neutrophils, and rare plasma cells. Occasional macrophages demonstrating hemophagocytic activity (arrow) are identified

After delivery, the patient continued to be febrile and was started on ampicillin, gentamicin, and clindamycin for a possible chorioamnionitis. CT angiogram was negative for PE, but showed a 20 cm liver with heterogeneous enhancement; no lymphadenopathy was seen. Sputum and bronchoscopy cultures were negative. On day 8, she remained febrile to >38.0°C, and the diagnosis of HLH was considered. Of note, there was no family history of HLH. Laboratories revealed a ferritin of 10 800 ng/mL (10‐107 ng/mL), increased from 2929 ng/mL on day 3, lactate dehydrogenase (LDH) 733 U/L (140‐271 U/L), increased from 409 on day 3, AST 736 U/L, triglycerides 707 mg/dL (<150 mg/dL). On day 9, bone marrow biopsy was without evidence of hemophagocytosis. Demonstration of hemophagocytes on bone marrow biopsy is helpful in making the diagnosis of HLH, but lack of hemophagocytes does not exclude the diagnosis. On day 11, biopsy of the liver did show evidence of hemophagocytosis (see Figure 1). Soluble IL‐2R (sIL‐2R) came back at 10 580 pg/mL (<1033 pg/mL). sIL‐2R levels of >2515 have a 100% sensitivity and 72.5% specificity for the diagnosis of HLH.^13^ She was started on dexamethasone with weekly etoposide per the HLH‐94 protocol.^5^ EBV viral load by PCR was 246 000 copies/mL via PCR. CMV viral load by PCR was positive at 104 161 IU/mL(<1600 IU/mL). She was negative for HIV, parvovirus, and influenza. She was started on antiviral therapy with ganciclovir. She clinically improved and was discharged home on day 52 to continue to receive etoposide in outpatient infusion (week 6 of HLH‐94).

Unfortunately, 3 days after discharge, she represented with fevers to 39.4°C. Of note, she had not been able to get the valganciclovir that had been prescribed at discharge. EBV viral titer had increased from undetectable on day 35 of her prior admission to 658 000 copies/mL by PCR upon readmission. On day 4 of readmission, laboratories were notable for LDH 1015 U/L and ferritin>15 000 ng/mL. Her fevers persisted. The HLH‐94 protocol was restarted, going back to twice‐weekly etoposide. She was given a dose of rituximab 375 mg/m^2 ^on day 15 of readmission for the EBV viremia.

Her clinical status was tenuous, requiring intensive care unit admission for supraventricular tachycardia, gastrointestinal bleeding from corticosteroid‐induced gastritis and thrombocytopenia, and hypoxic respiratory distress. She was severely neutropenic and was placed on both G‐CSF and GM‐CSF. On day 17, blood cultures from both PICC and peripheral sources grew multiple drug‐resistant organisms; this was the first time her cultures had been positive for bacteremia to date. Despite intensive antibiotic treatment, blood cultures remained persistently positive, and sepsis progressed to septic shock requiring multiple vasopressors. Granulocyte transfusions were considered, but declined by family given minimal likelihood of efficacy. She required mechanical intubation on day 20 due to acute respiratory distress syndrome. She passed on day 24 of her second admission due to ventricular fibrillation arrest.

## 3 DISCUSSION

Our patient developed HLH in the context of what was clinically thought to be AFLP at the time of her time of presentation. In retrospect, the liver biopsy and changes seen in the placenta were more consistent with HELLP. She had HSV1, EBV, and CMV viremias by PCR. It is unclear if one of these viremias was a driver of the HLH, or if the viremias were secondary to an immunocompromised state (although we suspect the latter). In our review of the literature, patients who develop HLH in the setting of pregnancy generally do well after delivery or termination of the pregnancy (Table 1). This, unfortunately, was not the case for our patient, perhaps suggesting that HLH that develops in the context of acute hepatic failure in pregnancy (either HELLP or AFLP) represents a subset of patients with a particularly poor prognosis. To this end, we found one available case report which describes a patient who developed HLH soon after an emergent C‐section for HELLP who was able to achieve a remission and went to allogenic bone marrow transplant. This patient unfortunately died of relapsed disease just under 1 year after transplant.^11^ Another patient who developed HLH in the setting of AFLP passed away due to complications of the HLH despite administration of etoposide‐based therapy, similar to our patient described here.^12^

Our review of published case reports also suggests that patients who develop HLH during pregnancy with associated EBV viremia may have poor outcomes. In our patient, the EBV reactivation was felt to be secondary to her immunocompromised state, but the other case reports we found did not describe an antecedent serious illness like the HELLP syndrome our patient had. Of the three case reports, we found pregnant patients with HLH and EBV viremia, only one patient was alive at last follow‐up. Of note, this patient was diagnosed much earlier in her pregnancy (11 weeks, compared to 29 and 30 weeks for the other cases in the literature and 35 weeks for our patient). Our patient, as well as those presented in the literature, received an etoposide‐based regimen. Our patient was also given antiviral medication, but perhaps more prompt administration of rituximab for the EBV would have improved her outcome. Neither of the patients described in other case reports who developed EBV viremia and HLH in the third trimester received rituximab.

Thus, in the very limited published experience, development of HLH in the setting of HELLP syndrome or AFLP as well as HLH that develops in the third trimester with associated EBV viremia appears to have a poor prognosis, even when compared to other situations in which patients develop HLH peripartum. We encourage other clinicians to present their cases in order to better understand if indeed HLH in association with AFLP/HELLP or EBV in pregnancy is unique situations with a poor prognosis requiring prompt recognition and perhaps treatment that is not etoposide‐based; our patient and others described did not achieve or did not have durable responses after etoposide‐based therapies. Our experience suggests that patients who fit diagnostic criteria for HLH during pregnancy should be checked for EBV viremia and prompt administration of antiviral therapy and rituximab should be employed if EBV titers are positive.

## CONFLICT OF INTEREST

The authors have no relevant conflict of interests.

## AUTHOR CONTRIBUTION

SS, YK, and EB: performed the literature search and wrote the manuscript. DF: provided pathology images. DC: helped provide clarification of the patient's infectious disease course.
